# Improving the performance of mutation-based evolving artificial neural networks with self-adaptive mutations

**DOI:** 10.1371/journal.pone.0307084

**Published:** 2024-07-15

**Authors:** Motoaki Hiraga, Masahiro Komura, Akiharu Miyamoto, Daichi Morimoto, Kazuhiro Ohkura

**Affiliations:** 1 Faculty of Mechanical Engineering, Kyoto Institute of Technology, Kyoto, Japan; 2 Graduate School of Advanced Science and Engineering, Hiroshima University, Hiroshima, Japan; 3 Department of Mechanical and Control Engineering, Kyushu Institute of Technology, Fukuoka, Japan; Sichuan University, CHINA

## Abstract

Neuroevolution is a promising approach for designing artificial neural networks using an evolutionary algorithm. Unlike recent trending methods that rely on gradient-based algorithms, neuroevolution can simultaneously evolve the topology and weights of neural networks. In neuroevolution with topological evolution, handling crossover is challenging because of the competing conventions problem. Mutation-based evolving artificial neural network is an alternative topology and weights neuroevolution approach that omits crossover and uses only mutations for genetic variation. This study enhances the performance of mutation-based evolving artificial neural network in two ways. First, the mutation step size controlling the magnitude of the parameter perturbation is automatically adjusted by a self-adaptive mutation mechanism, enabling a balance between exploration and exploitation during the evolution process. Second, the structural mutation probabilities are automatically adjusted depending on the network size, preventing excessive expansion of the topology. The proposed methods are compared with conventional neuroevolution algorithms using locomotion tasks provided in the OpenAI Gym benchmarks. The results demonstrate that the proposed methods with the self-adaptive mutation mechanism can achieve better performance. In addition, the adjustment of structural mutation probabilities can mitigate topological bloat while maintaining performance.

## Introduction

Neuroevolution is an approach within the field of machine learning that utilizes evolutionary algorithms to design artificial neural networks [1–3]. While recent trending methods commonly used in deep learning [4, 5] and deep reinforcement learning [6, 7] rely on gradient-based algorithms, neuroevolution optimizes neural networks through an evolutionary process involving natural selection and genetic variation. The main advantage of neuroevolution is that it utilizes population-based search methods, which enable broader exploration of the solution space and avoid being trapped in local optima. In addition, neuroevolution uses gradient-free optimization, making it applicable to problems in which the derivative information of the objective function is either unavailable or unreliable. Consequently, it can optimize or learn network structures, hyperparameters for controlling learning, or features of the algorithm itself, which are often challenging to address using gradient-based approaches [3, 8].

The most straightforward approaches to neuroevolution encode only the weight values as genotypes. These approaches have been successfully applied to various applications, including the design of controllers for robots [9, 10] and game-playing agents [11, 12]. However, the parameters that determine the network structure, such as the number of layers, the number of nodes in each layer, and the arrangement of recurrent connections, are regarded as hyperparameters. Designers must specify the network structure before evolution begins, which requires domain knowledge, intuition, and experimentation. On the other hand, Topology and Weight Evolving Artificial Neural Networks (TWEANNs) [13] evolve both the neural network structure and the weights simultaneously.

Examples of TWEANNs are GeNeralized Acquisition of Recurrent Links (GNARL) [14], Evolutionary Programming Network (EPNet) [15], Cellular Encoding (CE) [16], and Evolutionary Acquisition of Neural Topologies (EANT) [17, 18]. GNARL is an algorithm based on evolutionary programming that evolves the topology and weights of recurrent neural networks [14]. Similarly, EPNet utilizes an evolutionary programming algorithm that employs mutation operators to evolve the topology; however, the weights are modified only by a hybrid training algorithm based on backpropagation and simulated annealing [15]. CE employs indirect encoding, which can evolve using a genetic programming algorithm [16]. The encoding process uses a graph-based representation, referred to as a grammar tree, to define instructions for constructing neural networks. EANT evolves neural networks from a minimal structure using two optimization loops: structural exploration, which develops a new neural network structure, and structural exploitation, which adjusts the weights of the neural networks [17, 18].

NeuroEvolution of Augmenting Topologies (NEAT) [13] is the most popular and widely used TWEANN algorithm. In this algorithm, the evolution starts from a population of individuals with a minimal network structure and incrementally grows their topology. The NEAT algorithm uses a historical marker known as the innovation number, which tracks the ancestor of each gene. This number is assigned to a new gene whenever a node or connection is added through structural mutations. These innovation numbers are used to handle the speciation of the population and crossover between individuals with different topologies. To date, many successor algorithms, including state-of-the-art TWEANNs, have been developed based on NEAT [8, 19–23].

In most cases, managing crossover in neuroevolution is challenging due to the competing conventions problem [1, 13, 24, 25]. This problem arises when applying crossover between genotypes that are encoded differently but represent neural networks with similar behaviors. For example, when two parents have very different genotypes but exhibit high fitness, crossover between them may produce offspring with lower fitness. Moreover, in TWEANN algorithms, genome length varies depending on the network topology, which makes it challenging to define a consistent crossover between individuals. The NEAT algorithm mitigates the competing conventions problem using innovation numbers; however, crossover can still have disruptive effects [26].

As an alternative TWEANN approach, Mutation-Based Evolving Artificial Neural Network (MBEANN) [26] uses only mutations for genetic variation, omitting the use of crossover. In addition, structural mutations in MBEANN are designed not to affect the behavior of the phenotype to avoid fitness degradation. Moreover, the MBEANN algorithm introduces a novel encoding technique to define subnetworks within an individual as operons, and each operon grows independently throughout the evolution. Similar to NEAT, the individuals in MBEANN are initialized with a minimal structure. MBEANN outperformed NEAT in tasks such as the double-pole balancing problem [26, 27] and the automatic design of controllers for robotic swarms [28, 29].

Despite outperforming NEAT in various applications, MBEANN has limitations that must be addressed. One of the limitations is the difficulty in setting the hyperparameter known as the mutation step size, which controls the magnitude of changes in the weights and biases of neural networks. In MBEANN, structural mutations are designed to have small or no changes in the behavior of the neural networks. Consequently, exploration depends on the parameter mutation, which perturbs the weights and biases. The mutation step size in MBEANN is set to a fixed value [26–28]; therefore, a method for balancing the exploration-exploitation tradeoff is required.

Another limitation is that the network structure tends to bloat in MBEANN. Individuals in MBEANN are expected to generate modularized subnetworks, each of which corresponds to an operon in the genotype. Each operon is designed to evolve independently without generating connections between two different operons. Moreover, each operon is structurally mutated with a constant probability; therefore, individuals with many operons have a higher expectation of applying structural mutations, leading to an exponential expansion in the network topology relative to the number of operons [28, 30]. This rapid growth is beneficial when neural networks require many nodes and connections to perform a task. However, overgrown neural networks typically incur high computational costs.

This study proposes two novel improvements to the MBEANN algorithm to overcome the existing limitations.

- *Self-adaptation of the mutation step size.* A self-adaptive mutation mechanism, which is often used in evolution strategies [31, 32], is employed to automatically adjust the mutation step size during the evolution process. The mutation step size is coevolved within each individual, enabling automatic balancing between exploration and exploitation.
- *Adjustment of structural mutation probabilities.* The structural mutation probabilities are normalized depending on the size of the neural networks, which can expand the network topology at a speed similar to that of the traditional MBEANN in the early stages and gradually decrease the growth rate as the structure matures. This improvement aims to evolve high-performance neural networks with topologies smaller than those generated by the conventional MBEANN algorithm.

In this study, these improvements are compared and discussed with the traditional MBEANN and NEAT using the HalfCheetah-v4 and Ant-v4 locomotion tasks provided in OpenAI Gym [33, 34].

The remainder of this paper is organized as follows. The section “Mutation-based evolving artificial neural network” provides an introduction to MBEANN with details on the genetic encoding method and mutation operators. The section “Self-adaptive mutations for MBEANN” describes the proposed methods. The section “Experimental setup” explains the locomotion tasks provided in Open AI Gym and describes the algorithm settings. The section “Results and discussion” provides the results of the experiments with a discussion. Finally, the last section concludes the paper.

## Mutation-based evolving artificial neural network

The Mutation-Based Evolving Artificial Neural Network (MBEANN) [26] is a TWEANN approach that uses only mutations for genetic variation. In TWEANN algorithms, it is difficult to handle crossover because of the competing conventions problem [1, 13, 24, 25]. Therefore, inspired by asexual reproduction, the MBEANN algorithm omits crossover to generate offspring. The remainder of this section describes the genetic encoding method and mutation operators used in the MBEANN algorithm.

### Genetic encoding

**Fig 1:**
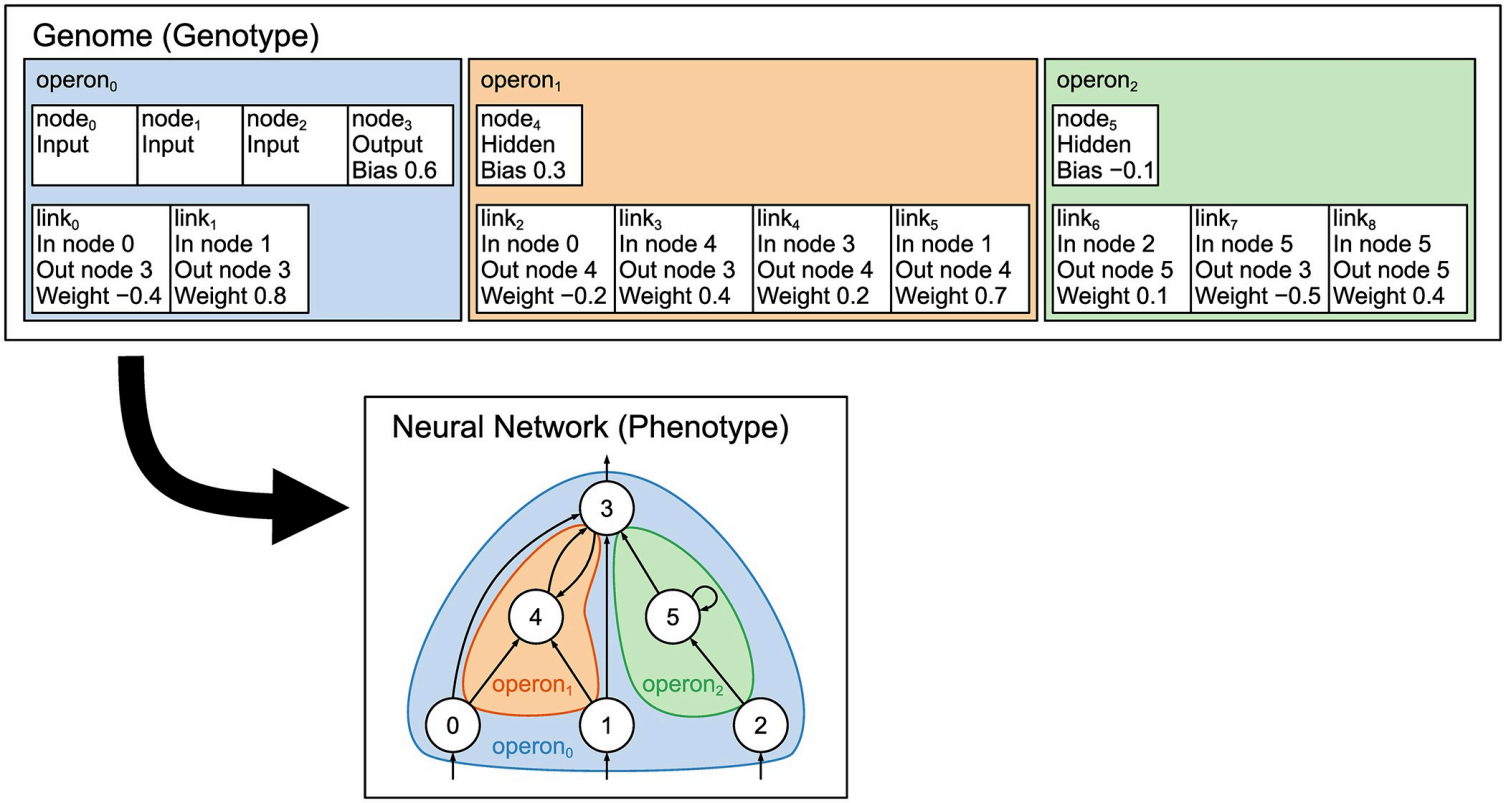
Example of the genotype-phenotype mapping in MBEANN. A genome consists of operons, each of which corresponds to a subnetwork within the neural network. In this example, the genome of the neural network consists of three operons, that is, operon_0_, operon_1_, and operon_2_. Note that operon_0_ includes only nodes from the input and output layers, along with the direct connections between them. When the add-node mutation is applied to operon_0_, a new operon is created using the added hidden node.

A genome in the MBEANN algorithm is designed to contain sets of genes called operons. An example of the genotype-phenotype mapping in the MBEANN algorithm is shown in Fig 1. Each operon contains a set of node genes and a set of link genes. Each node gene has an identification number, a node type indicating the layer to which it belongs, and a bias value. The bias value is used to shift the activation function of the corresponding node; therefore, it is assigned only to the hidden and output nodes. Each link gene provides an identification number, identification numbers of the in-node and out-node, and a weight value. The genome in the MBEANN algorithm is described as follows:

$$\begin{array}{c} \text{genome}=\{{\text{operon}}_{0},{\text{operon}}_{1},\cdots ,{\text{operon}}_{m}\}, \end{array}$$ (1)

$$\begin{array}{c} {\text{operon}}_{i}=\left\{{\text{node}}_{j}|j\in O_{Ni}\right\}\cup \left\{{\text{link}}_{k}|k\in O_{Li}\right\}, \end{array}$$ (2)

where *m* is the number of operons added to the genome, node_*j*_ is the node gene with an identification number of *j*, link_*k*_ is the link gene with an identification number of *k*, and *O*_*Ni*_ and *O*_*Li*_ are the set of identification numbers of nodes and links in operon_*i*_, respectively.

In the same way as the NEAT algorithm, MBEANN starts with minimally structured neural networks and incrementally expands their topology. The genomes in the initial population have only one operon, that is, operon_0_. The nodes in the input and output layers are assigned to operon_0_. The operon_0_ is unique because it can contain only nodes in the input and output layers and direct connections between them. New operons are generated by adding hidden nodes via the add-node mutation (as explained later). Therefore, operon_*i*_(*i* ≠ 0) consists of one or more hidden nodes, including connections between nodes within operon_*i*_ and connections bridging nodes between operon_*i*_ and operon_0_. It should be noted that the connections between operon_*i*_ and operon_*j*_(*i* ≠ *j*, *i* ≠ 0, *j* ≠ 0) are prohibited. Thus, operons grow independently from each other, which allows the neural network to have modularized subnetworks.

### Mutation operators

The MBEANN algorithm evolves neural networks by mutating both the weights and the structure of the network. There are two types of structural mutations: add-node and add-connection mutations. To prevent decreases in fitness values due to changes in the network structure, these mutations are designed to be nearly or completely neutral. In other words, the behavior of neural networks before and after structural mutations exhibits small or no changes. In addition, the weights and biases are mutated by applying small random values. The mutation operators are described as follows.

#### Add-node mutation

**Fig 2:**
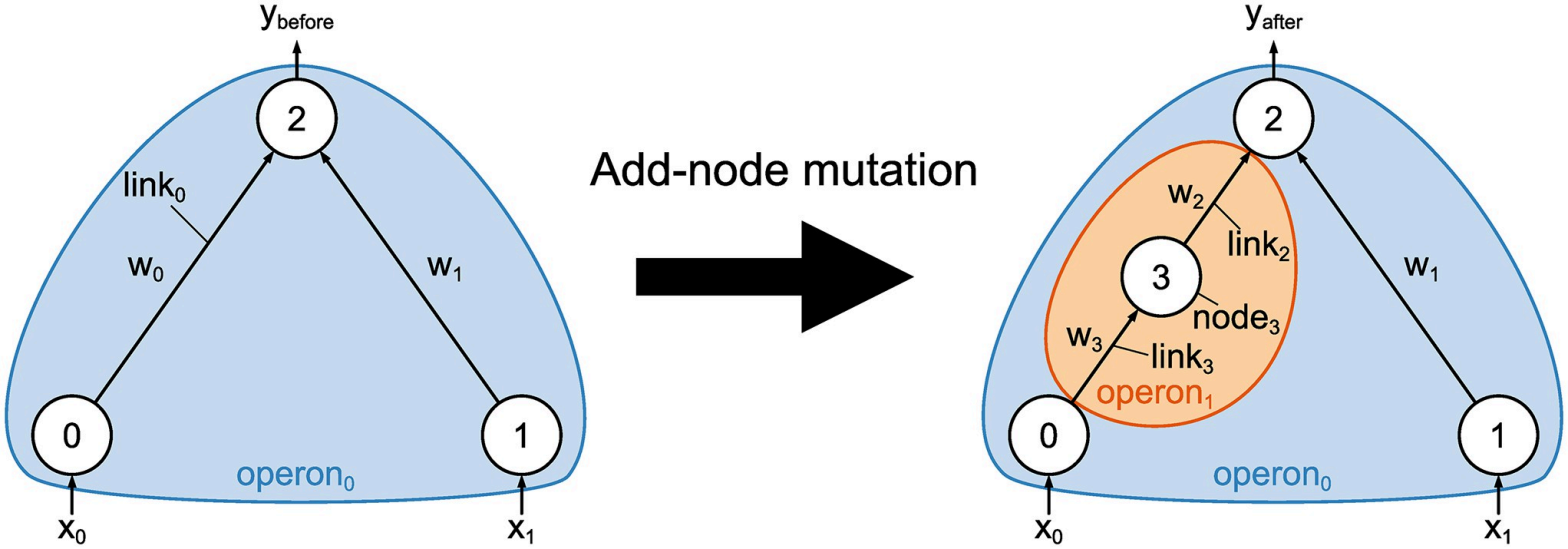
Example of the add-node mutation. In this figure, the connection of link_0_, which has the weight value of *w*_0_, is selected and replaced with node_3_, link_2_, and link_3_. If the selected connection to be replaced belongs to operon_0_, a new operon is generated with the new node and connections.

The add-node mutation replaces the existing connection with a new node and two connections. This mutation is applied to each operon with a probability of *p*_node_. If this mutation is applied to operon_0_, a new operon is generated with the new node and connections. The pseudo-code of the add-node mutation is described in Algorithm 1, and an example of this mutation is illustrated in Fig 2.

**Algorithm 1:** Pseudo-code of the add-node mutation.

**1** **foreach** operon in genome **do**

**2**  **if** rand(0, 1)<*p*_node_ **then**

**3**     ▹ rand(0,1) is a uniform random number between 0 and 1

**4**   Randomly select one connection from the operon;

**5**   **if** selected connection is in operon_0_ **then**

**6**    Remove the selected connection;

**7**    Create a new operon with a new node and two new connections;

**8**   **else**

**9**    Remove the selected connection;

**10**    Add a new node and two new connections to the current operon;

**11**   **end**

**12**  **end**

**13** **end**

The structural mutations of MBEANN are designed to minimize the effects on the behavior of the neural networks. For example, in Fig 2, the add-node mutation should be designed to satisfy *y*_before_ ≃ *y*_after_. Let link_*i*_ have a weight value of *w*_*i*_ and let the activation function *φ* be applied to the hidden and output nodes. Then, the equation to be satisfied is described as *w*_0_*x*_0_ ≃ *w*_2_*φ*(*w*_3_*x*_0_). Assume that *w*_0_ = *w*_2_ and *φ*(*x*) = 1/(1 + *e*^*α*(*β* − *x*)^). The error due to the mutation, denoted as *L*(*x*_0_), can be defined as follows:

$$\begin{array}{c} L(x_{0})=|x_{0}-\varphi \left(w_{3}x_{0}\right)|=|x_{0}-\frac{1}{1+e^{\alpha (\beta -w_{3}x_{0})}}|. \end{array}$$ (3)

Here, 0 < *φ*(*x*) < 1, and assuming that the inputs take values within the range [0, 1], the parameters of the activation function are set to have *α* = 4.63/*w*_3_ and *β* = 0.5*w*_3_ (*w*_3_ ≠ 0) to minimize the integral of Eq 3 [28]. For simplicity, the weight value of the new connection is set to *w*_3_ = 1. The parameter *β* behaves as a bias that shifts the midpoint of the sigmoid activation function. Therefore, the bias value of the newly added node is initialized with *β* = 0.5.

#### Add-connection mutation

**Fig 3:**
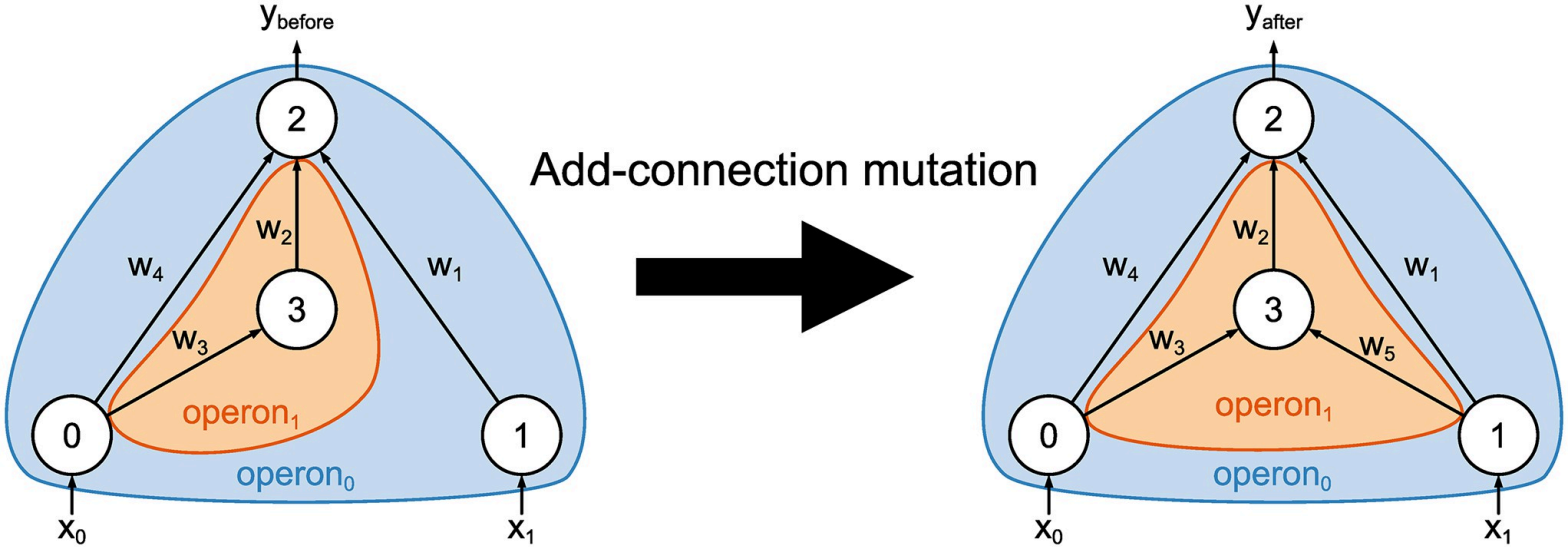
Example of the add-connection mutation. A new connection with the weight value of *w*_5_ is created from node_1_ to node_3_. The nodes to be connected are selected either from two nodes within the same operon or from one in operon_0_ and the other in the operon being mutated. The weight value of the new connection is set to zero.

The add-connection mutation generates a new connection that connects two previously unconnected nodes. This mutation is applied to each operon with a probability of *p*_link_. The pseudo-code of the add-connection mutation is given in Algorithm 2, and an example is shown in Fig 3. When this mutation is applied to operon_*i*_, a new connection is generated to connect two nodes within operon_*i*_ or one each from operon_*i*_ and operon_0_. Connections between operon_*i*_ and operon_*j*_(*i* ≠ *j*, *i* ≠ 0, *j* ≠ 0) are prohibited. Therefore, operons grow independently of each other and generate modularized subnetworks. The weight value of the newly added connection is set to zero to maintain the mutation neutral.

**Algorithm 2:** Pseudo-code of the add-connection mutation.

**1** **foreach** operon in genome **do**

**2**  **if** rand(0, 1) < *p*_link_ **then**

**3**   Randomly select one node from the operon;

**4**   Randomly select another node from the current operon or from operon_0_;

**5**   Add a new connection between them with a weight value of 0;

**6**  **end**

**7** **end**

#### Parameter mutation

Each weight and bias value is perturbed by adding a random value with a probability of *p*_param_. When the parameter mutation is applied to the link_*i*_ with the weight value of *w*_*i*_, the weight value is mutated to $w_{i}^{\prime }$ using the following equation:

$$\begin{array}{c} w_{i}^{\prime }=w_{i}+\varsigma \cdot N_{i}\left(0,1\right), \end{array}$$ (4)

where *ς* is the step size that controls the magnitude of the mutation and *N*_*i*_(0, 1) is a random value sampled from the standard normal distribution for each variable *i*. The bias values are also mutated using Eq 4.

## Self-adaptive mutations for MBEANN

The parameter mutation in the conventional MBEANN algorithm uses a constant mutation step size [26–28]. In the proposed method, the step size adapts its value during the evolution process. The self-adaptation of a mutation step size is often used in evolution strategies [31, 32]. In the MBEANN algorithm, the number of dimensions of the search space dynamically changes depending on the network topology. Therefore, it is difficult to construct the covariance matrix often used in state-of-the-art evolution strategies [35, 36] or to use a set of mutation step sizes [31, 32].

The self-adaptive mutation for MBEANN is implemented using an approach based on self-adaptive evolution strategies with one step size [30–32]. Each individual has a single mutation step size shared within it to mutate its weights and biases. The step size is updated once per generation before applying the parameter mutation. The self-adaptive parameter mutation is defined as follows:

$$\begin{array}{c} \varsigma^{\prime }=\varsigma \cdot e^{\tau \cdot N(0,1)}, \end{array}$$ (5)

$$\begin{array}{c} w_{i}^{\prime }=w_{i}+\varsigma^{\prime }\cdot N_{i}\left(0,1\right), \end{array}$$ (6)

where *ς*′ is the updated mutation step size and *N*(0, 1) is a random value sampled from the standard normal distribution. The value *τ* is the learning parameter that determines the rate and precision of self-adaptation. Usually, in evolution strategies, it is chosen to have a value of $\tau =\kappa /\sqrt{n}$, where *n* is the dimension of the search space and *κ* is a constant value often set to have *κ* = 1 [31]. In the proposed method, the parameters are set to *κ* = 1 and *n* with the total number of weights and biases in the genome.

In addition, a method for adjusting the probabilities of structural mutations is proposed in this study. In the conventional MBEANN algorithm, add-node and add-connection mutations are applied to each operon with a corresponding constant probability. Thus, an individual with many operons has a higher expectation of adding nodes and connections, resulting in an exponential growth of the network topology with an increase in the number of operons [28, 30]. This characteristic is beneficial if neural networks require many nodes and connections to perform tasks. However, overgrown neural networks are computationally expensive, and their behavior is generally difficult to analyze and interpret.

In the proposed method, the structural mutation probabilities are normalized by the number of operons. Structural mutation is applied to each operon with a normalized mutation probability $\hat{p}$, which is defined as follows:

$$\begin{array}{c} \hat{p}=1-{\left(1-\bar{p}\right)}^{\frac{1}{M}}, \end{array}$$ (7)

where *M* is the number of operons in the individual, and $\bar{p}$ is the probability of applying the structural mutation at least once within the individual. The $\hat{p}$ and $\bar{p}$ are defined independently for add-node and add-connection mutations. With the normalized mutation probabilities, ${\hat{p}}_{\text{node}}$ is used instead of *p*_node_ in Algorithm 1 and ${\hat{p}}_{\text{link}}$ is used instead of *p*_link_ in Algorithm 1. By setting a constant probability for $\bar{p}$, an individual with many operons has a lower probability of mutating each operon. In addition, a lower bound is set for $\hat{p}$ to prevent stagnation of the topological evolution.

## Experimental setup

**Fig 4:**
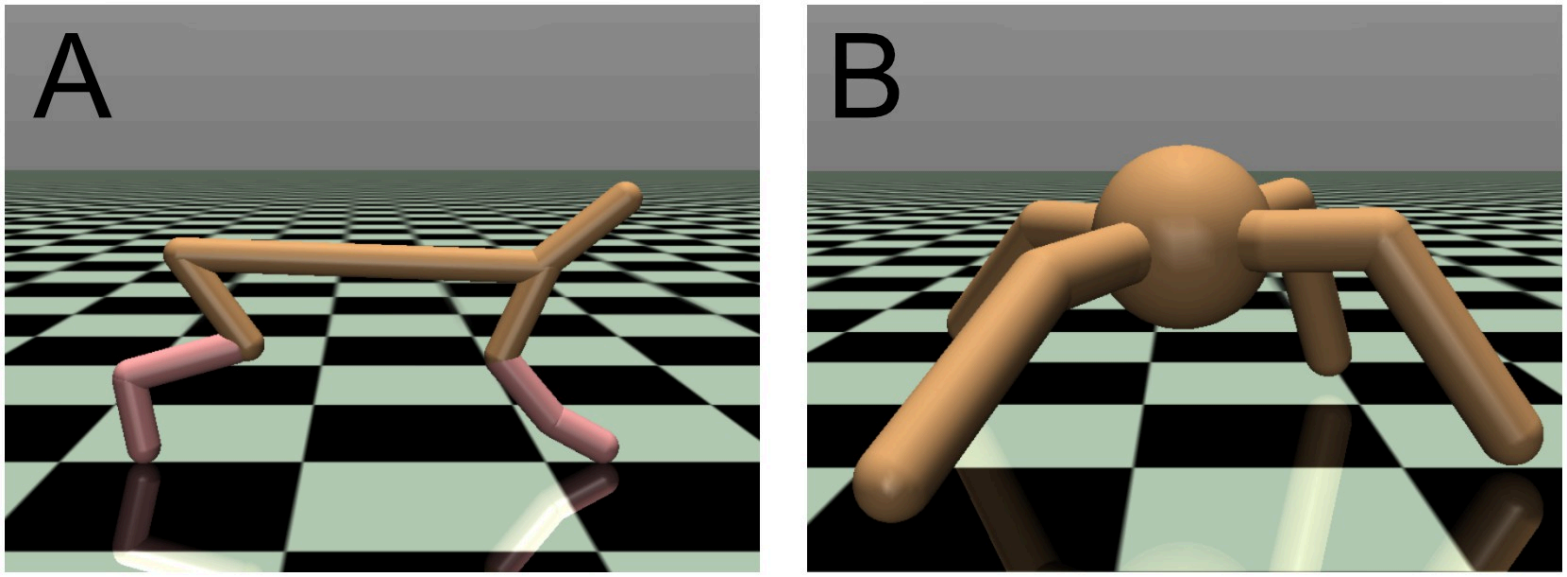
Screenshots of (A) HalfCheetah-v4 and (B) Ant-v4 provided in OpenAI Gym using the MuJoCo physics simulator.

To compare the algorithms, the HalfCheetah-v4 and Ant-v4 locomotion tasks provided in OpenAI Gym [33, 34] with the MuJoCo physics engine [37] were employed as benchmark tasks (see Fig 4). The default parameter settings were employed for HalfCheetah-v4 and Ant-v4. OpenAI Gym is an open-source library that provides a collection of benchmarks for developing and comparing reinforcement learning algorithms. The following are belief explanations for the HalfCheetah-v4 and Ant-v4 tasks. For details, see the Gym library documentation [33, 34].

### HalfCheetah-v4

HalfCheetah-v4 employs a two-dimensional robot with two legs, each of which has three hinge joints. The goal of this task is to make the robot move forward as quickly as possible. The observation space consists of the position, velocity, angle, and angular velocity of various body parts of the robot. Seventeen values in the observation space are fed into the neural network. The action space has six values corresponding to the torque applied to each hinge joint. The values for the action space are determined based on the outputs of the neural network. The reward *r* is defined as follows:

$$\begin{array}{c} r=f-0.1c, \end{array}$$ (8)

where *f* is the reward for moving forward and *c* is the control cost that penalizes when the robot takes actions that are too large. The episode is truncated when the episode length exceeds 1000 time steps.

### Ant-v4

Ant-v4 employs a four-legged robot, with each leg having two hinge joints. Similar to HalfCheetah-v4, the goal is to make the robot move forward as quickly as possible. The observation space consists of the position, velocity, angle, and angular velocity of various body parts, whereas the action space consists of the torques applied to the hinge joints. There are twenty-seven values in the observation space and eight in the action space. The reward *r* is defined as follows:

$$\begin{array}{c} r=h+f-0.5c, \end{array}$$ (9)

where *h* is the healthy reward given when the robot is stable and balanced, *f* is the forward reward that encourages forward progress, and *c* is the control cost that penalizes actions that are too large. The robot is said to be unhealthy when any of the state values is no longer finite or the *z*-coordinate of the torso is not within the range [0.2, 1.0]. The episode ends when the robot is unhealthy or the episode length reaches 1000 time steps.

### Algorithm settings

The proposed methods were compared with conventional TWEANN algorithms, that is, NEAT [13] and the standard MBEANN [26]. The NEAT algorithm was implemented using the neat-python library [38] and the standard MBEANN using pyMBEANN [39]. To enhance clarity, the proposed algorithm that uses the self-adaptive mutation step size is referred to as SA-MBEANN. Simultaneously, the proposed method with the self-adaptive mutation step size and normalized structural mutation probabilities is denoted as SANP-MBEANN. Both SA-MBEANN and SANP-MBEANN were developed using the pyMBEANN library.

For all algorithms, the sum of the rewards obtained within the episode was used as the fitness value of the individual. This cumulative reward within the episode was also used as an indicator to evaluate and compare the algorithms. The population sizes were set to 200 and 500 for HalfCheetah-v4 and Ant-v4, respectively. The maximum number of generations was set to 100 for HalfCheetah-v4 and 200 for Ant-v4. These settings are due to the task difficulty, that is, Ant-v4 is more challenging than HalfCheetah-v4.

In the family of MBEANN algorithms, tournament selection was employed, as described in the original MBEANN [26]. The best individual was selected from a tournament size of 20 for HalfCheetah-v4 and 50 for Ant-v4. The mutation probabilities were set to *p*_node_ = 0.03, *p*_link_ = 0.3, and *p*_param_ = 1.0 based on previous studies [27–29]. In the standard MBEANN, the step size of the parameter mutation was set to *ς* = 0.01 for HalfCheetah-v4 and *ς* = 0.005 for Ant-v4. In the self-adaptive mutation, the step size was initialized with the same values as the standard MBEANN. The step size in the self-adaptive mutation adapts its value during the evolution process using Eq 5.

To prevent stagnation of the evolution and to avoid large leaps in the parameter mutation, a constraint was set for the step size, limiting it to the range within [0.001, 0.1]. For SANP-MBEANN, the mutation probabilities were normalized using Eq 7, with the hyperparameters set to ${\bar{p}}_{\text{node}}=0.03$ and ${\bar{p}}_{\text{link}}=0.3$. By setting the probabilities ${\bar{p}}_{\text{node}}$ and ${\bar{p}}_{\text{link}}$ to the same value as *p*_node_ and *p*_link_ in the standard MBEANN, the network structure in the proposed method is assumed to grow similarly to that of the standard MBEANN in the early evolutionary generations, and the mutation probabilities gradually decrease as the network growth progresses. The lower bound for ${\hat{p}}_{\text{node}}$ was set to 0.01 and ${\hat{p}}_{\text{link}}$ to 0.1.

The NEAT algorithm also incorporates mutations for adding nodes and connections, as well as for perturbing weights and biases. The mutation probabilities were set to have similar values with MBEANN, i.e., 0.03 for adding a new node, 0.3 for adding a new link, and 1.0 for perturbing weights and biases. The mutation step size (called the mutation power in neat-python) was set to the same value as that of the standard MBEANN. The other parameters of NEAT were determined based on the examples in the neat-python library [38]. In particular, the compatibility coefficient of the disjoint and excess genes was set to 1.0, and the coefficient of weights and biases was set to 1.0. The compatibility threshold was set to 3.0. The survival threshold, which determines the proportion of each species surviving as parents to the next generation, was set to 0.2. A sigmoid function was employed for the activation function. However, with these settings, the performance of NEAT was relatively poor and unstable. Therefore, elitism selection was added to NEAT with a size of 20 for HalfCheetah-v4 and 50 for Ant-v4.

The full lists of the parameter values used in the experiments are summarized in S1 Text.

## Results and discussion

**Fig 5:**
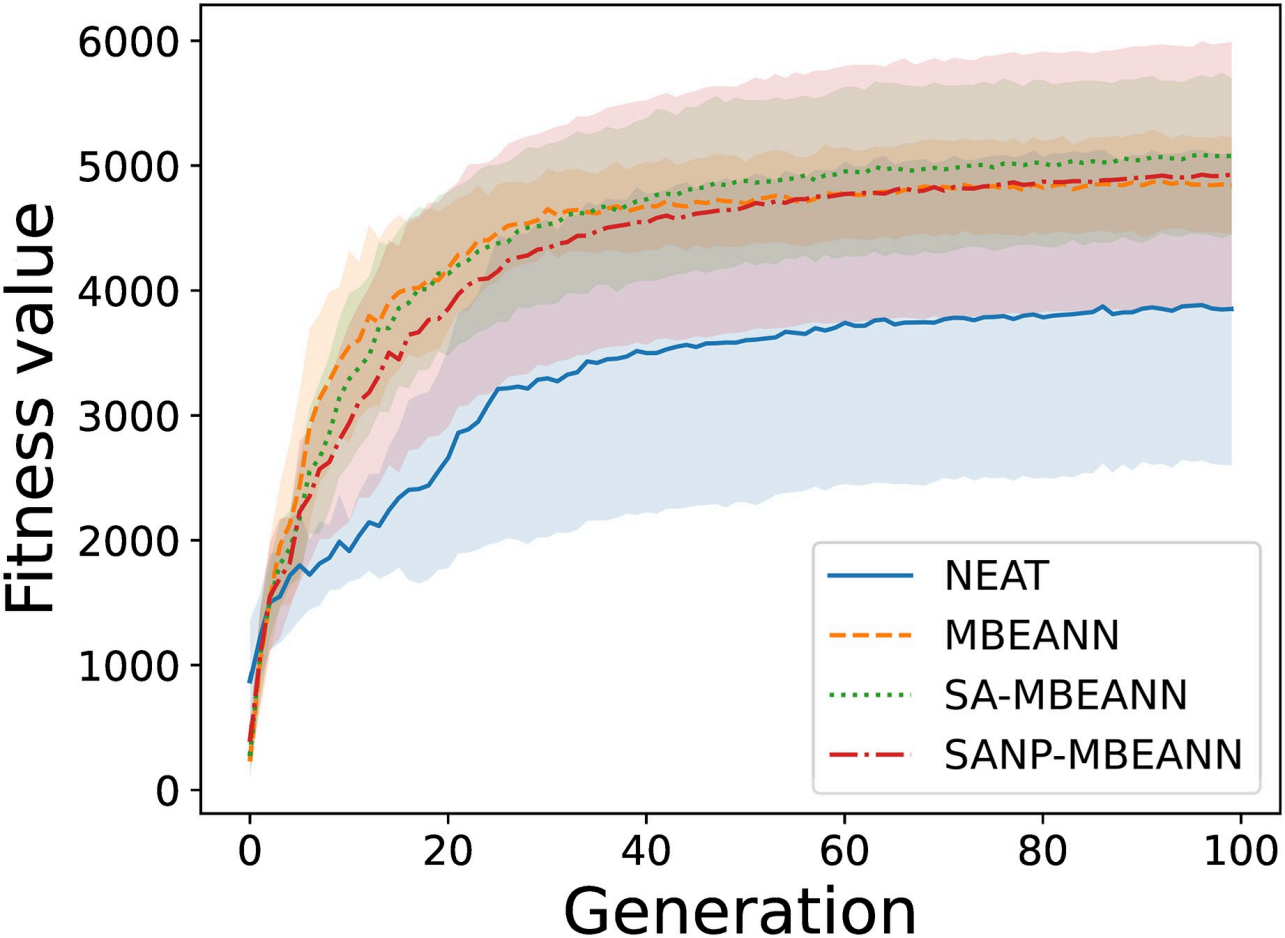
Transitions of the fitness value of the best individual in HalfCheetah-v4. Each line represents the mean of the best fitness values over 15 trials, and the shaded regions around them indicate the standard deviations.

**Fig 6:**
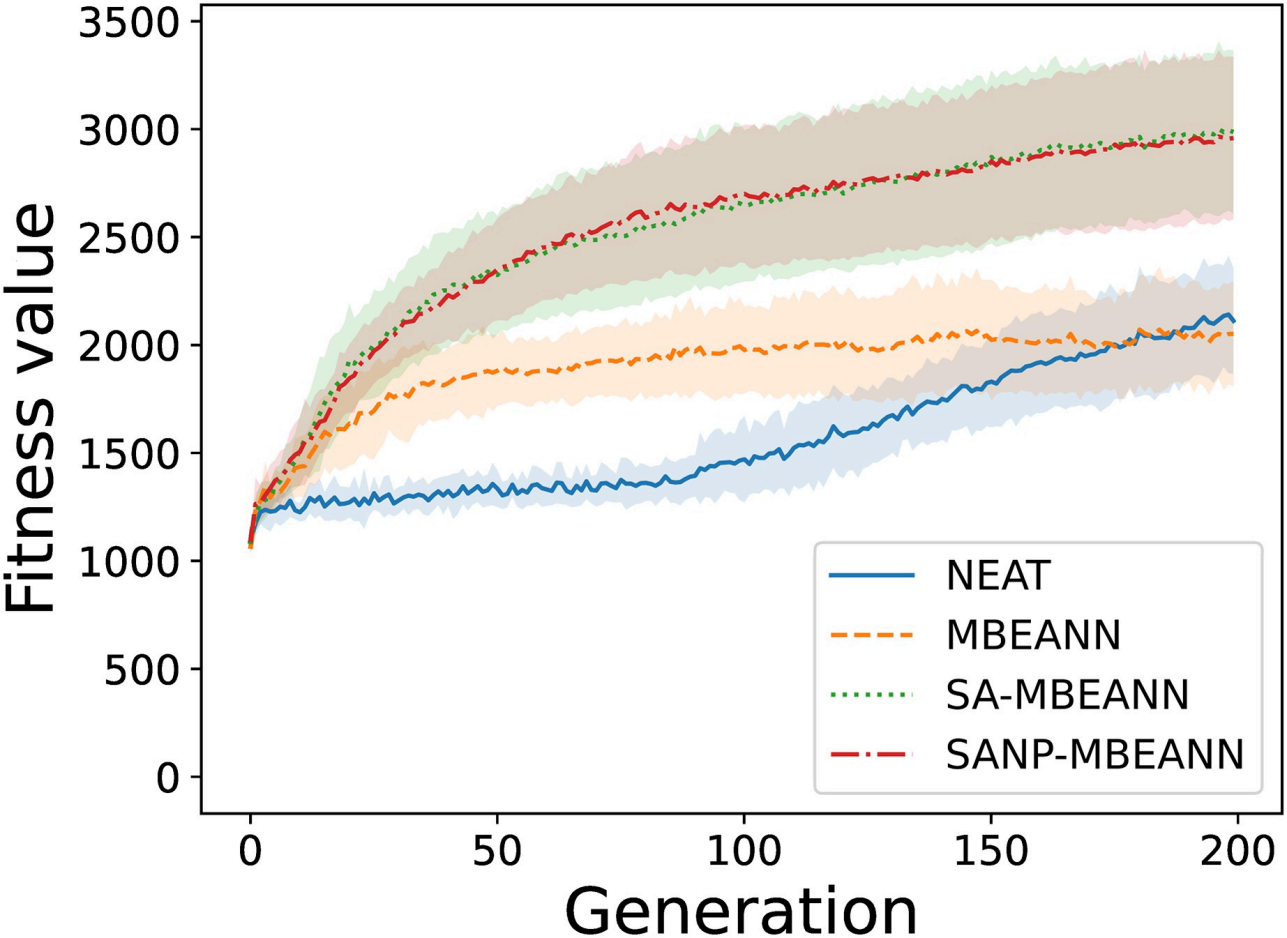
Transitions of the fitness value of the best individual in Ant-v4. Each line represents the mean of the best fitness values over 15 trials, and the shaded regions around them indicate the standard deviations.

Fifteen evolutionary trials were conducted for each algorithm. Figs 5 and 6 show the fitness transitions of the best individual within the population in HalfCheetah-v4 and Ant-v4, respectively. In HalfCheetah-v4, all MBEANN algorithms (MBEANN, SA-MBEANN, and SANP-MBEANN) exhibited relatively similar fitness transitions, with SA-MBEANN stagnating at slightly higher values. For each pair of algorithms, a statistical test was performed using data from the last generation to compare the fitness values to which each algorithm converged. The two-sided Mann-Whitney *U* test with Bonferroni correction was performed at a significance level of 0.05. Significant differences were found between NEAT and each MBEANN algorithm (Bonferroni-corrected *p* < 0.05; henceforth, all *p*-values are Bonferroni-corrected). No significant differences were observed between the MBEANN algorithms (*p* > 0.05). In Ant-v4, SA-MBEANN and SANP-MBEANN obtained fitness values higher than those of the standard MBEANN and NEAT (*p* < 0.05). No significant differences were observed between NEAT and MBEANN or between SA-MBEANN and SANP-MBEANN (*p* > 0.05). When comparing SA-MBEANN and SANP-MBEANN, SANP-MBEANN obtained slightly lower fitness values than SA-MBEANN in HalfCheetah-v4; however, in both environments, they showed similar fitness transitions, as shown in Figs 5 and 6.

**Fig 7:**
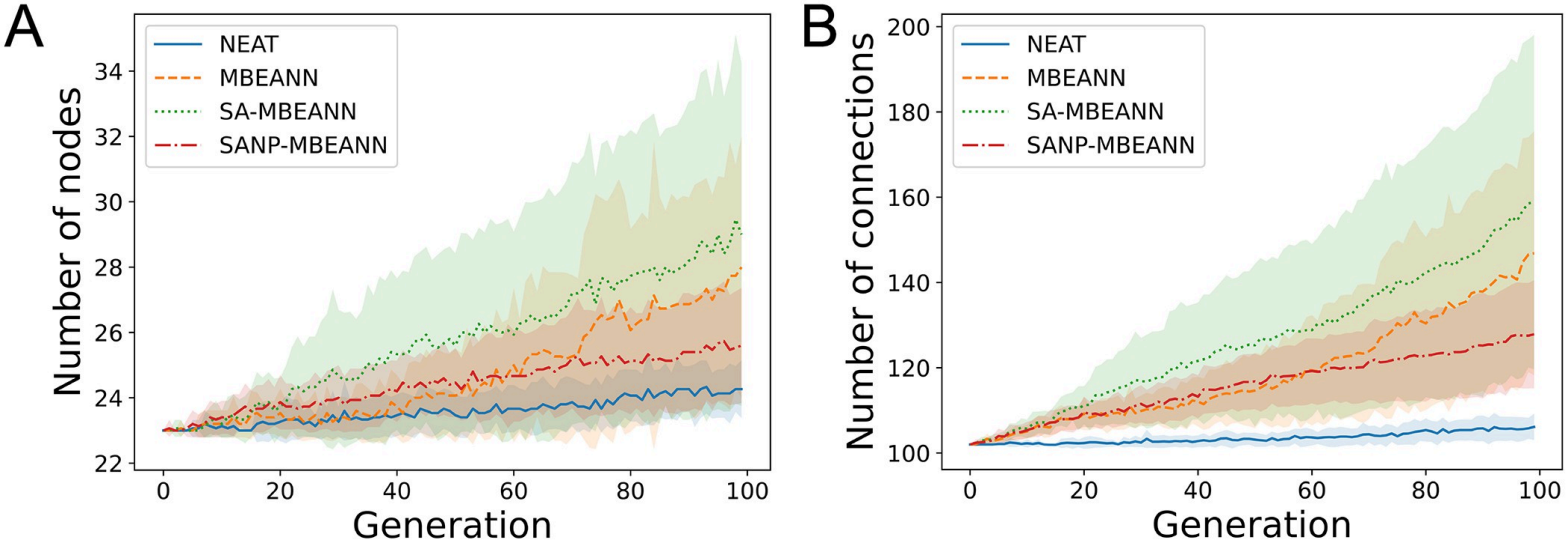
Transitions of the network structure of the best individual in HalfCheetah-v4. (A) Transitions of the number of nodes in the individual, including 17 input and 6 output nodes. (B) Transitions of the number of connections. Each line represents the mean over 15 trials, and the shaded regions around them indicate the standard deviations.

**Fig 8:**
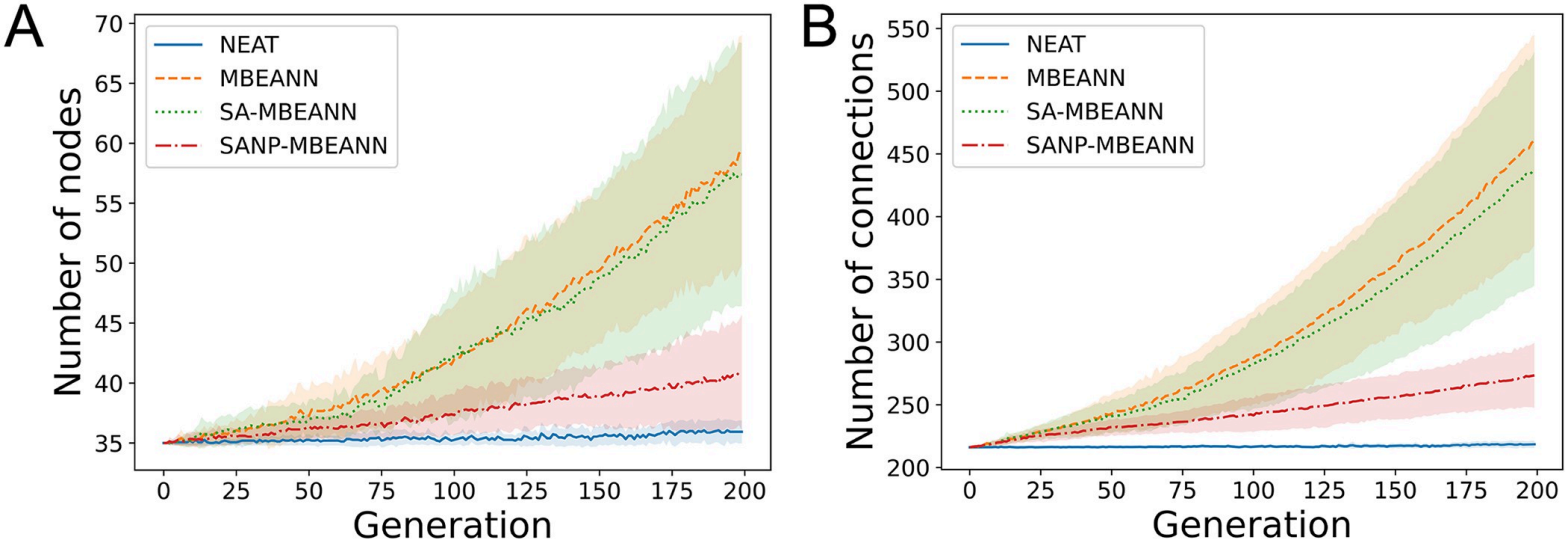
Transitions of the network structure of the best individual in Ant-v4. (A) Transitions of the number of nodes in the individual, including 27 input and 8 output nodes. (B) Transitions of the number of connections. Each line represents the mean over 15 trials, and the shaded regions around them indicate the standard deviations.

The algorithms used in this study also evolve the structure of neural networks. Figs 7 and 8 show the transitions of the number of nodes and connections of the best individuals in HalfCheetah-v4 and Ant-v4, respectively. The two-sided Mann-Whitney *U* test with Bonferroni correction was performed for each pair of algorithms using data from the last generation. The MBEANN and SA-MBEANN algorithms showed a similar tendency in the network structure transitions because they have the same structural mutation probabilities. No significant differences were observed between MBEANN and SA-MBEANN in either HalfCheetah-v4 or Ant-v4 (*p* > 0.05). Notably, in Ant-v4, MBEANN and SA-MBEANN showed exponential growth in the network structure, as shown in Fig 8. The growth of the network structures in the NEAT algorithm was very slow compared to the MBEANN family in both HalfCheetah-v4 and Ant-v4. Significant differences were found between NEAT and each MBEANN algorithm (*p* < 0.05), except for the number of nodes between NEAT and SANP-MBEANN in HalfCheetah-v4 (*p* > 0.05). This exception is because SANP-MBEANN automatically adjusts the structural mutation probabilities, which results in a smaller network topology. Indeed, the network structure of SANP-MBEANN grew more linearly than those of MBEANN and SA-MBEANN, as can be seen in Figs 7 and 8.

SANP-MBEANN is expected to grow similarly to the standard MBEANN in the early evolutionary generations, and the mutation probabilities gradually decrease as the topology expands. Therefore, in HalfCheetah-4, which has only 100 generations, no significant differences were observed between MBEANN and SANP-MBEANN, or between SA-MBEANN and SANP-MBEANN (*p* > 0.05). However, in Ant-v4, significant differences were observed between MBEANN and SANP-MBEANN, and between SA-MBEANN and SANP-MBEANN (*p* < 0.05). Because SA-MBEANN and SANP-MBEANN showed similar fitness transitions, as shown in Figs 5 and 6, these results imply that SANP-MBEANN can mitigate bloats in network structures while maintaining performance.

**Fig 9:**
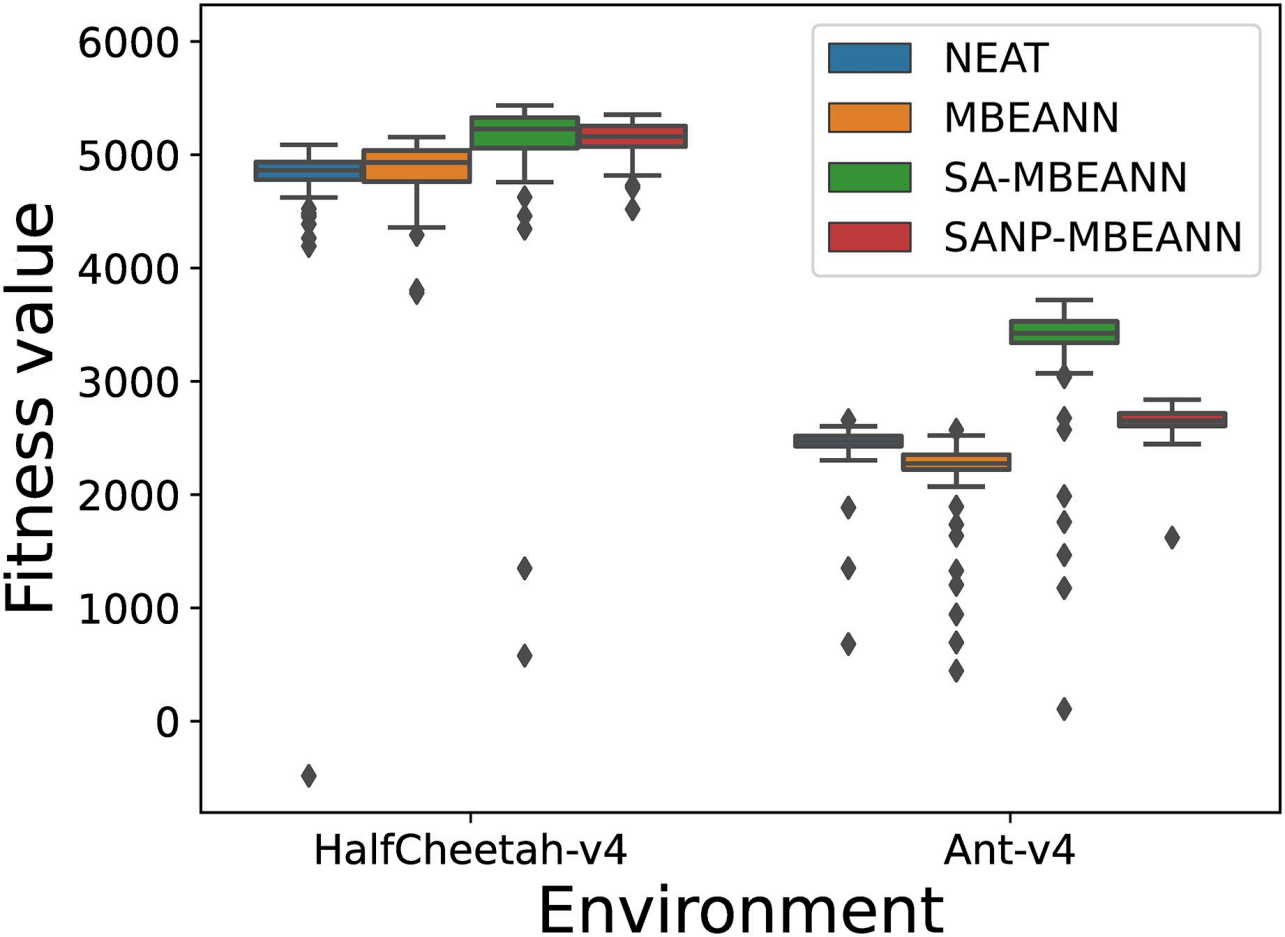
Results of the re-evaluation for 100 trials using the best-evolved individuals.

For further discussion, the individual that obtained the highest fitness value throughout the evolution was used for re-evaluation. The best-evolved individual from each algorithm was re-evaluated for 100 trials. Fig 9 shows the results of the re-evaluation. The two-sided Mann-Whitney *U* test with Bonferroni correction was performed for each pair of algorithms. In HalfCheetah-v4, the proposed methods SA-MBEANN and SANP-MBEANN obtained significantly higher performance than NEAT and MBEANN (*p* < 0.05). There was no significant difference between SA-MBEANN and SANP-MBEANN (*p* > 0.05). In Ant-v4, statistically significant differences were observed between all pairs of algorithms (*p* < 0.05). Moreover, SA-MBEANN significantly outperformed the other algorithms in Ant-v4. Both SA-MBEANN and SANP-MBEANN significantly outperformed the standard MBEANN, as shown in Fig 9.

**Fig 10:**
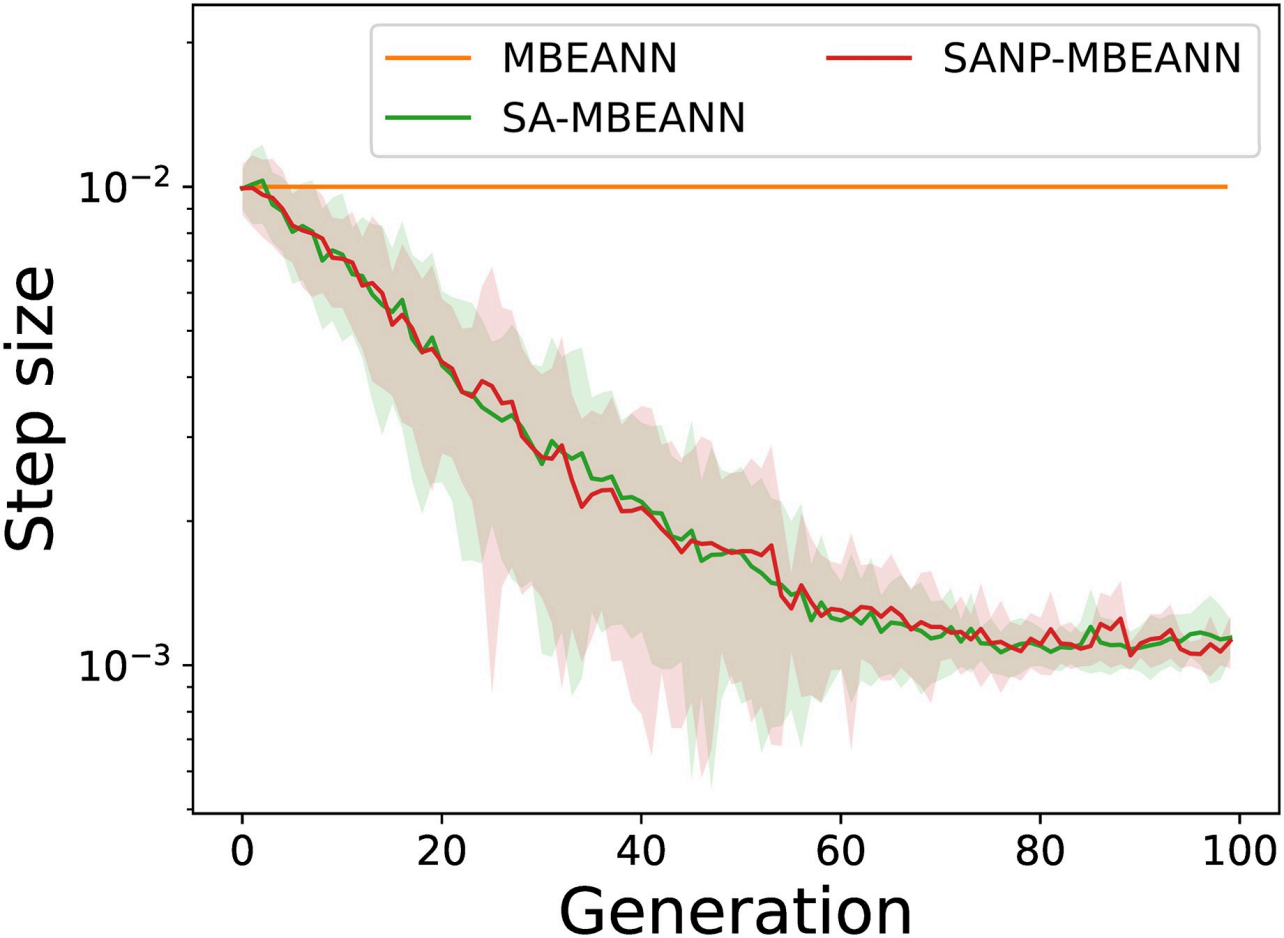
Transitions of the step size of the best individual in HalfCheetah-v4. Each line represents the mean over 15 trials, while the shaded regions around them show the standard deviations.

**Fig 11:**
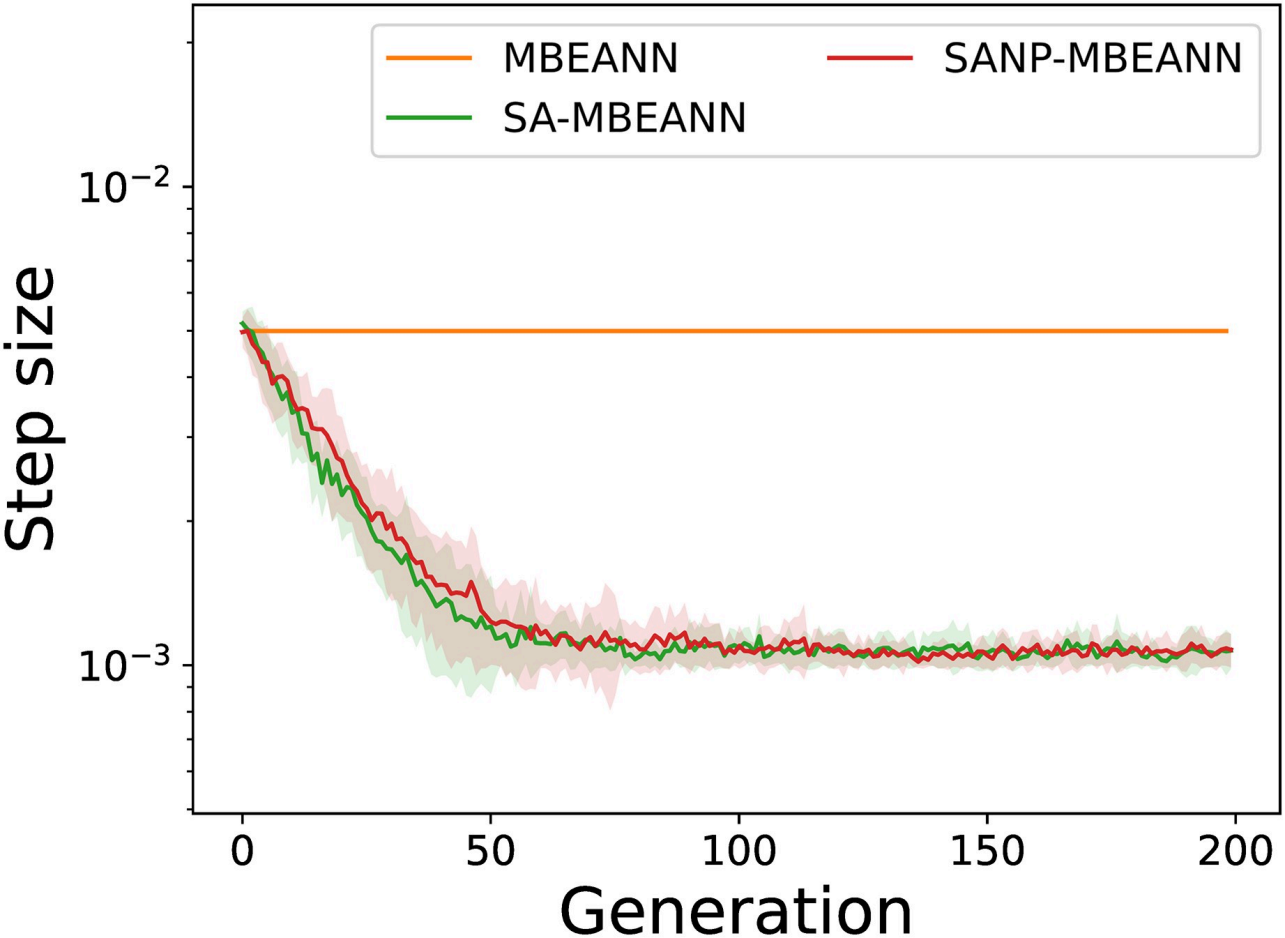
Transitions of the step size of the best individual in Ant-v4. Each line represents the mean over 15 trials, while the shaded regions around them show the standard deviations.

The MBEANN algorithms with self-adaptation of the mutation step size (SA-MBEANN and SANP-MBEANN) achieved higher performance. Figs 10 and 11 show the step size transitions of the best individuals in HalfCheetah-v4 and Ant-v4, respectively. As shown in Figs 10 and 11, the mutation step size gradually decreased during evolution. The self-adaptive mutation mechanism facilitates a balance between exploration and exploitation. In particular, if larger changes in weights and biases lead to better solutions, individuals with larger mutation step sizes are more likely to survive. Conversely, individuals with smaller step sizes improve their performance based on the current solutions. As the evolutionary process shifts from exploration to exploitation, the mutation step size asymptotically decreases, enabling the algorithm to converge to an optimal solution. Therefore, it can be assumed that the self-adaptation mechanism automatically adjusts the mutation step size to refine the solutions, resulting in better performance.

Overall, the MBEANN algorithms with the self-adaptive mutation mechanism (SA-MBEANN and SANP-MBEANN) achieved better performance than the standard MBEANN. In addition, the proposed approach with normalized mutation probabilities (SANP-MBEANN) showed better performance than the standard MBEANN with smaller neural network structures. Therefore, the proposed approach with normalized mutation probabilities (SANP-MBEANN) is the best choice when considering the computational cost. However, when comparing SA-MBEANN and SANP-MBEANN, SA-MBEANN achieved better performance in Ant-v4, as shown in Fig 9. Considering the task difficulty, Ant-v4 might require more complex structured neural networks, which were not sufficiently evolved in SANP-MBEANN.

The limitation of the proposed approach is the lack of optimization of the neural network structures. In the proposed method, the structure of neural networks continues to grow as evolution progresses. Therefore, if the evolutionary run is executed for many generations, the structure of the neural networks continuously grows over time, eventually reaching the limit of the computational cost. However, suppressing topological evolution may lead to low performance by constraining the behavior of neural networks. Finding an optimal structure is a challenging problem, not only for the proposed approach but also for other TWEANN algorithms, as well as for the recently emerged research topic known as neural architecture search [40, 41]. Approaches for determining the optimal structure of neural networks and guiding topological evolution toward this optimal structure are left for future research.

## Conclusion

In conclusion, this study has demonstrated the effectiveness of two improvements to the MBEANN algorithm. First, the self-adaptive mutation mechanism was integrated into MBEANN to automatically adjust the mutation step size, which was used to control the magnitude of perturbation in the weights and biases of the neural networks. Second, the structural mutation probabilities were normalized depending on the size of the neural networks to avoid overgrowth of the network topology. The results of this study showed that MBEANN with the self-adaptive mutation mechanism outperformed conventional algorithms by dynamically balancing exploration and exploitation. In addition, the proposed method with self-adaptive mutation and normalized structural mutation probabilities achieved better performance than the standard MBEANN with smaller network structures.

In tasks that require complex structured neural networks, the normalized structural mutation probabilities are assumed to suppress topological evolution, which may degrade performance. Further research will explore novel approaches for topological optimization in MBEANN, with an emphasis on devising mechanisms to identify optimal network structures and guide topological evolution toward them.

## Supporting information

S1 Text The document describes the parameter settings of the algorithms used in this study. In addition, the results of the Mann-Whitney *U* tests are listed.
(PDF)

S1 File CSV files used to generate Figs 5–11. (ZIP)

## References

[1] Floreano D, Dürr P, Mattiussi C. Neuroevolution: from architectures to learning. Evolutionary Intelligence. 2008;1:47–62. doi: 10.1007/s12065-007-0002-4

[2] Yao X. Evolving artificial neural networks. Proceedings of the IEEE. 1999;87(9):1423–1447. doi: 10.1109/5.784219

[3] Stanley KO, Clune J, Lehman J, Miikkulainen R. Designing neural networks through neuroevolution. Nature Machine Intelligence. 2019;1(1):24–35. doi: 10.1038/s42256-018-0006-z

[4] LeCun Y, Bengio Y, Hinton G. Deep learning. Nature. 2015;521(7553):436–444. doi: 10.1038/nature14539 26017442

[5] Goodfellow I, Bengio Y, Courville A. Deep learning. MIT Press; 2016.

[6] Mnih V, Kavukcuoglu K, Silver D, Rusu AA, Veness J, Bellemare MG, et al. Human-level control through deep reinforcement learning. Nature. 2015;518(7540):529–533. doi: 10.1038/nature14236 25719670

[7] Sutton RS, Barto AG. Reinforcement learning: an introduction. MIT Press; 2018.

[8] Miikkulainen R, Liang J, Meyerson E, Rawal A, Fink D, Francon O, et al. Evolving deep neural networks. In: Artificial Intelligence in the Age of Neural Networks and Brain Computing. Elsevier; 2024. pp. 269–287.

[9] Nolfi S, Floreano D. Evolutionary robotics: the biology, intelligence, and technology of self-organizing machines. MIT Press; 2000.

[10] Trianni V. Evolutionary swarm robotics: evolving self-organising behaviours in groups of autonomous robots. Springer; 2008.

[11] Salimans T, Ho J, Chen X, Sidor S, Sutskever I. Evolution strategies as a scalable alternative to reinforcement learning. arXiv:1703.03864 [Preprint]. 2017. Available from: 10.48550/arXiv.1703.03864.

[12] Such FP, Madhavan V, Conti E, Lehman J, Stanley KO, Clune J. Deep neuroevolution: genetic algorithms are a competitive alternative for training deep neural networks for reinforcement learning. arXiv:1712.06567 [Preprint]. 2017. Available from: 10.48550/arXiv.1712.06567.

[13] Stanley KO, Miikkulainen R. Evolving neural networks through augmenting topologies. Evolutionary Computation. 2002;10(2):99–127. doi: 10.1162/106365602320169811 12180173

[14] Angeline PJ, Saunders GM, Pollack JB. An evolutionary algorithm that constructs recurrent neural networks. IEEE Transactions on Neural Networks. 1994;5(1):54–65. doi: 10.1109/72.265960 18267779

[15] Yao X, Liu Y. A new evolutionary system for evolving artificial neural networks. IEEE Transactions on Neural Networks. 1997;8(3):694–713. doi: 10.1109/72.572107 18255671

[16] Gruau F. Neural network synthesis using cellular encoding and the genetic algorithm. PhD thesis, Ecole Normale Supirieure de Lyon, France; 1994.

[17] Kassahun Y, Sommer G. Efficient reinforcement learning through evolutionary acquisition of neural topologies. In: Proceeding of the 13th European Symposium on Artificial Neural Networks; 2005. pp. 259–266.

[18] Siebel NT, Sommer G. Evolutionary reinforcement learning of artificial neural networks. International Journal of Hybrid Intelligent Systems. 2007;4(3):171–183. doi: 10.3233/HIS-2007-4304

[19] Papavasileiou E, Cornelis J, Jansen B. A systematic literature review of the successors of “neuroevolution of augmenting topologies”. Evolutionary Computation. 2021;29(1):1–73. doi: 10.1162/evco_a_00282 33151100

[20] Gaier A, Ha D. Weight agnostic neural networks. Advances in Neural Information Processing Systems. 2019;32.

[21] Showalter I, Schwartz HM. Neuromodulated multiobjective evolutionary neurocontrollers without speciation. Evolutionary Intelligence. 2021;14(4):1415–1430. doi: 10.1007/s12065-020-00394-9

[22] Jaafar SAB, Suzuki R, Komori S, Arita T. Effects of excessive elitism on the evolution of artificial creatures with NEAT. Artificial Life and Robotics. 2024;29(2):286–297. doi: 10.1007/s10015-024-00948-5

[23] Khamesian S, Malek H. Hybrid self-attention NEAT: a novel evolutionary self-attention approach to improve the NEAT algorithm in high dimensional inputs. Evolving Systems. 2024;15:489–503. doi: 10.1007/s12530-023-09510-3

[24] Schaffer JD, Whitley D, Eshelman LJ. Combinations of genetic algorithms and neural networks: a survey of the state of the art. In: Proceedings of the International Workshop on Combinations of Genetic Algorithms and Neural Networks (COGANN-92). IEEE; 1992. pp. 1–37.

[25] Radcliffe NJ. Genetic set recombination and its application to neural network topology optimisation. Neural Computing & Applications. 1993;1:67–90. doi: 10.1007/BF01411376

[26] Ohkura K, Yasuda T, Kawamatsu Y, Matsumura Y, Ueda K. MBEANN: mutation-based evolving artificial neural networks. In: Advances in Artificial Life. Springer; 2007. pp. 936–945.

[27] Hiraga M, Watanabe Y, Ohkura K. TWEANN approach to the double pole balancing problem: feature comparison between NEAT and MBEANN [in Japanese]. Transactions of the Institute of Systems, Control and Information Engineers. 2022;35(5):126–132. doi: 10.5687/iscie.35.126

[28] Hiraga M, Ohkura K. Topology and weight evolving artificial neural networks in cooperative transport by a robotic swarm. Artificial Life and Robotics. 2022;27(2):324–332. doi: 10.1007/s10015-021-00716-9

[29] Katada Y, Hirokawa T, Hiraga M, Ohkura K. MBEANN for robotic swarm controller design and the behavior analysis for cooperative transport. Journal of Robotics and Mechatronics. 2023;35(4):997–1006. doi: 10.20965/jrm.2023.p0997

[30] Komura M, Miyamoto A, Hiraga M, Morimoto D, Ohkura K. Proposal and evaluation of surrogate-assisted self-adaptive MBEANN [in Japanese]. Transactions of the Institute of Systems, Control and Information Engineers. Forthcoming 2024;37(8):216–224.

[31] Beyer HG, Schwefel HP. Evolution strategies: a comprehensive introduction. Natural Computing. 2002;1:3–52. doi: 10.1023/A:1015059928466

[32] Eiben AE, Smith JE. Introduction to evolutionary computing. Springer; 2015.

[33] Brockman G, Cheung V, Pettersson L, Schneider J, Schulman J, Tang J, et al. OpenAI gym. arXiv:1606.01540 [Preprint]. 2016. Available from: 10.48550/arXiv.1606.01540.

[34] Towers M, Terry JK, Kwiatkowski A, Balis JU, Cola Gd, Deleu T, et al. Gymnasium; 2023. Available from: https://zenodo.org/record/8127025.

[35] Hansen N, Ostermeier A. Completely derandomized self-adaptation in evolution strategies. Evolutionary Computation. 2001;9(2):159–195. doi: 10.1162/106365601750190398 11382355

[36] Hansen N. The CMA evolution strategy: a comparing review. Towards a New Evolutionary Computation: Advances in the Estimation of Distribution Algorithms. 2006;192:75–102. doi: 10.1007/3-540-32494-1_4

[37] Todorov E, Erez T, Tassa Y. MuJoCo: a physics engine for model-based control. In: 2012 IEEE/RSJ International Conference on Intelligent Robots and Systems. IEEE; 2012. pp. 5026–5033.

[38] McIntyre A, Kallada M, Miguel CG, Feher de Silva C, Netto ML. neat-python; 2017. Available from: https://github.com/CodeReclaimers/neat-python.

[39] Hiraga M. pyMBEANN; 2023. Available from: https://github.com/motoHiraga/pyMBEANN.

[40] Zoph B, Le QV. Neural architecture search with reinforcement learning. In: International Conference on Learning Representations; 2017.

[41] Elsken T, Metzen JH, Hutter F. Neural architecture search: a survey. Journal of Machine Learning Research. 2019;20(55):1–21.

