# Supplementary material for: Improving the performance of mutation-based evolving artificial neural networks with self-adaptive mutations
Source: PLoS One. 2024 Jul 15;19(7):e0307084. doi: 10.1371/journal.pone.0307084 (PMC11249216; doi:10.1371/journal.pone.0307084)
Supplement: S1 Text — In addition, the results of the Mann-Whitney U tests are listed. (PDF) [file pone.0307084.s001.pdf]

Improving the performance of mutation-based evolving artificial neural networks with self-adaptive mutations: Supporting information

Motoaki Hiraga[1*], Masahiro Komura[2], Akiharu Miyamoto[2], Daichi Morimoto[3], Kazuhiro Ohkura[2*]

1 Faculty of Mechanical Engineering, Kyoto Institute of Technology, Kyoto, Japan
2 Graduate School of Advanced Science and Engineering, Hiroshima University, Hiroshima, Japan
3 Department of Mechanical and Control Engineering, Kyushu Institute of Technology, Fukuoka, Japan

* hiraga@kit.ac.jp (MH), kohkura@hiroshima-u.ac.jp (KO)

Abstract

The “Parameter settings” section provides the parameter settings of the algorithms used in the study. In addition, the “Results of the Mann-Whitney U tests” section provides the results of the statistical tests.

Parameter settings

The parameters common to all algorithms are listed in Table A. The parameters to control selection and mutation in MBEANN, SA-MBEANN, and SANP-MBEANN are listed in Table B. The parameters of the mutation step size are listed in Table C. Table D lists the parameters used for NEAT. See the neat-python documentation for further details of each parameter [1]. The other parameters not listed in Tables A or D take the default values defined in the neat-python library. In Tables A–D , the values that differ between HalfCheetah-v4 and Ant-v4 are shown in bold font.

Table A. Settings common to all algorithms (MBEANN, SA-MBEANN, SANP-MBEANN, and NEAT).

Parameter	HalfCheetah-v4	Ant-v4
Population size	**200**	**500**
Maximum number of generations	**100**	**200**
Number of inputs	**17**	**27**
Number of outputs	**6**	**8**
Number of hidden nodes in the initial population	0	0
Connections in the initial population	fully connected	fully connected
Initialization type of the weight and bias values	gaussian	gaussian
Mean of the weight and bias initialization	0	0
Standard deviation of the weight and bias initialization	**0.01**	**0.005**
Lower and upper bounds for the weight and bias values	$[-1.0, 1.0]$	$[-1.0, 1.0]$
Allow to generate recurrent connections	True	True
Activation function	sigmoid	sigmoid

Table B. Parameter settings for the selection and mutation in MBEANN, SA-MBEANN, and SANP-MBEANN.

Algorithm	Parameter	HalfCheetah-v4	Ant-v4
All MBEANN algorithms	Tournament size	**20**	**50**
	Parameter mutation probability p_{param}	1.0	1.0
Standard MBEANN/SA-MBEANN	Add-node mutation probability p_{node}	0.03	0.03
	Add-connection mutation probability p_{link}	0.3	0.3
SANP-MBEANN	Add-node mutation probability $\bar{p}_{\text{node}}$	0.03	0.03
	Add-connection mutation probability $\bar{p}_{\text{link}}$	0.3	0.3
	Lower bound for the normalized add-node mutation probability $\hat{p}_{\text{node}}$	0.01	0.01
	Lower bound for the normalized add-connection mutation probability $\hat{p}_{\text{link}}$	0.1	0.1

Table C. Parameter settings of the mutation step size in MBEANN, SA-MBEANN, and SANP-MBEANN.

Algorithm	Parameter	HalfCheetah-v4	Ant-v4
Standard MBEANN	Mutation step size	**0.01**	**0.005**
SA-MBEANN/SANP-MBEANN	Initial mutation step size	**0.01**	**0.005**
	Lower and upper bounds for the mutation step size	$[0.001, 0.1]$	$[0.001, 0.1]$

Table D. Parameter settings used for the implementation of NEAT using the neat-python library.

Section	Parameter	HalfCheetah-v4	Ant-v4
[NEAT] section	No fitness termination	True	True
	Reset on extinction	False	False
[DefaultReproduction] section	Elitism	**20**	**50**
	Survival threshold	0.2	0.2
	Minimum species size	1	1
[DefaultSpeciesSet] section	Compatibility threshold	3.0	3.0
[DefaultGenome] section	Compatibility disjoint coefficient	1.0	1.0
	Compatibility weight coefficient	1.0	1.0
	Add node probability	0.03	0.03
	Delete node probability	0.0	0.0
	Add connection probability	0.3	0.3
	Delete connection probability	0.0	0.0
	Weight and bias mutation probability	1.0	1.0
	Weight and bias mutation power	**0.005**	**0.01**
	Weight and bias replace probability	0.0	0.0
	Default activation function	sigmoid	sigmoid
	Activation function mutation probability	0.0	0.0
	Default aggregation function	sum	sum
	Aggregation function mutation probability	0.0	0.0
	Mean of the initial response multiplier	1.0	1.0
	Standard deviation of the initial response multiplier	0.0	0.0
	Response multiplier mutation probability	0.0	0.0
	Response multiplier replace probability	0.0	0.0
[DefaultStagnation] section	Species fitness function	max	max
	Maximum stagnation generations	15	15
	Species elitism	4	4

Results of the Mann-Whitney U tests

In this study, two-sided Mann-Whitney U tests were performed using the SciPy library [2]. The Bonferroni correction was applied to reduce the likelihood of obtaining false-positive results (Type I errors) when performing multiple statistical comparisons. Tables E–K show the results of the Mann-Whitney U tests along with Bonferroni-corrected p-values using the data in Figs 5–9. The data (CSV files) used to generate figures and tables are provided in the supporting information "S1 File."

Table E. Results of the Mann-Whitney U tests for the fitness values in the last generation in Fig 5.

Algorithms	Test statistic U	p-value	Bonferroni-corrected p-value
NEAT vs MBEANN	41.0	0.003	0.019
NEAT vs SA-MBEANN	29.0	< 0.001	0.003
NEAT vs SANP-MBEANN	42.0	0.004	0.022
MBEANN vs SA-MBEANN	66.0	0.056	0.338
MBEANN vs SANP-MBEANN	68.0	0.068	0.408
SA-MBEANN vs SANP-MBEANN	110.0	0.934	1.000

Table F. Results of the Mann-Whitney U tests for the fitness values in the last generation in Fig 6.

Algorithms	Test statistic U	p-value	Bonferroni-corrected p-value
NEAT vs MBEANN	124.0	0.648	1.000
NEAT vs SA-MBEANN	1.0	< 0.001	< 0.001
NEAT vs SANP-MBEANN	8.0	< 0.001	< 0.001
MBEANN vs SA-MBEANN	6.0	< 0.001	< 0.001
MBEANN vs SANP-MBEANN	6.0	< 0.001	< 0.001
SA-MBEANN vs SANP-MBEANN	117.0	0.868	1.000

Table G. Results of the Mann-Whitney U tests for the number of nodes in the last generation in Fig 7A.

Algorithms	Test statistic U	p-value	Bonferroni-corrected p-value
NEAT vs MBEANN	41.5	0.003	0.016
NEAT vs SA-MBEANN	45.5	0.005	0.029
NEAT vs SANP-MBEANN	60.0	0.027	0.161
MBEANN vs SA-MBEANN	102.0	0.676	1.000
MBEANN vs SANP-MBEANN	145.5	0.172	1.000
SA-MBEANN vs SANP-MBEANN	144.5	0.188	1.000

Table H. Results of the Mann-Whitney U tests for the number of connections in the last generation in Fig 7B.

Algorithms	Test statistic U	p-value	Bonferroni-corrected p-value
NEAT vs MBEANN	0.0	< 0.001	< 0.001
NEAT vs SA-MBEANN	5.0	< 0.001	< 0.001
NEAT vs SANP-MBEANN	10.0	< 0.001	< 0.001
MBEANN vs SA-MBEANN	91.0	0.383	1.000
MBEANN vs SANP-MBEANN	153.0	0.097	0.579
SA-MBEANN vs SANP-MBEANN	167.0	0.025	0.150

Table I. Results of the Mann-Whitney U tests for the number of nodes in the last generation in Fig 8A.

Algorithms	Test statistic U	p-value	Bonferroni-corrected p-value
NEAT vs MBEANN	0.0	< 0.001	< 0.001
NEAT vs SA-MBEANN	2.5	< 0.001	< 0.001
NEAT vs SANP-MBEANN	9.5	< 0.001	< 0.001
MBEANN vs SA-MBEANN	121.0	0.740	1.000
MBEANN vs SANP-MBEANN	219.5	< 0.001	< 0.001
SA-MBEANN vs SANP-MBEANN	202.5	< 0.001	0.001

Table J. Results of the Mann-Whitney U tests for the number of connections in the last generation in Fig 8B.

Algorithms	Test statistic U	p-value	Bonferroni-corrected p-value
NEAT vs MBEANN	0.0	< 0.001	< 0.001
NEAT vs SA-MBEANN	0.0	< 0.001	< 0.001
NEAT vs SANP-MBEANN	0.0	< 0.001	< 0.001
MBEANN vs SA-MBEANN	121.5	0.724	1.000
MBEANN vs SANP-MBEANN	225.0	< 0.001	< 0.001
SA-MBEANN vs SANP-MBEANN	208.5	< 0.001	< 0.001

Table K. Results of the Mann-Whitney U tests for the data in Fig 9.

Environment	Algorithms	Test statistic U	p-value	Bonferroni-corrected p-value
HalfCheetah-v4	NEAT vs MBEANN	3792.0	0.003	0.019
	NEAT vs SA-MBEANN	1109.0	< 0.001	< 0.001
	NEAT vs SANP-MBEANN	697.0	< 0.001	< 0.001
	MBEANN vs SA-MBEANN	1579.0	< 0.001	< 0.001
	MBEANN vs SANP-MBEANN	1310.0	< 0.001	< 0.001
	SA-MBEANN vs SANP-MBEANN	5897.0	0.028	0.171
Ant-v4	NEAT vs MBEANN	9098.0	< 0.001	< 0.001
	NEAT vs SA-MBEANN	499.0	< 0.001	< 0.001
	NEAT vs SANP-MBEANN	574.0	< 0.001	< 0.001
	MBEANN vs SA-MBEANN	477.0	< 0.001	< 0.001
	MBEANN vs SANP-MBEANN	126.0	< 0.001	< 0.001
	SA-MBEANN vs SANP-MBEANN	9377.0	< 0.001	< 0.001

References

1. McIntyre A, Kallada M, Miguel CG, Feher de Silva C, Netto ML. neat-python; 2017. Available from: `https://github.com/CodeReclaimers/neat-python`.

2. Virtanen P, Gommers R, Oliphant TE, Haberland M, Reddy T, Cournapeau D, et al. SciPy 1.0: fundamental algorithms for scientific computing in Python. Nature Methods. 2020;17:261–272.
